# Risk factors for revision of primary total hip arthroplasty: a systematic review

**DOI:** 10.1186/1471-2474-13-251

**Published:** 2012-12-15

**Authors:** Julian JZ Prokopetz, Elena Losina, Robin L Bliss, John Wright, John A Baron, Jeffrey N Katz

**Affiliations:** 1 Brigham and Women’s Hospital, Harvard Medical School, Boston, MA, USA; 2 Harvard Medical School, Boston, MA, USA; 3 Boston University School of Public Health, Boston, MA, USA; 4 Veristat, Inc., Holliston, MA, USA; 5 University of North Carolina, Chapel Hill, NC, USA; 6 Harvard School of Public Health, Boston, MA, USA; 7 Orthopedic and Arthritis Center for Outcomes Research, Brigham and Women’s Hospital, 75 Francis St. OBC-4, Boston, MA, 02115, USA

**Keywords:** Total hip arthroplasty, Revision, Failure, Risk factor, Aseptic loosening, Infection, Dislocation, Systematic review

## Abstract

**Background:**

Numerous papers have been published examining risk factors for revision of primary total hip arthroplasty (THA), but there have been no comprehensive systematic literature reviews that summarize the most recent findings across a broad range of potential predictors.

**Methods:**

We performed a PubMed search for papers published between January, 2000 and November, 2010 that provided data on risk factors for revision of primary THA. We collected data on revision for any reason, as well as on revision for aseptic loosening, infection, or dislocation. For each risk factor that was examined in at least three papers, we summarize the number and direction of statistically significant associations reported.

**Results:**

Eighty-six papers were included in our review. Factors found to be associated with revision included younger age, greater comorbidity, a diagnosis of avascular necrosis (AVN) as compared to osteoarthritis (OA), low surgeon volume, and larger femoral head size. Male sex was associated with revision due to aseptic loosening and infection. Longer operating time was associated with revision due to infection. Smaller femoral head size was associated with revision due to dislocation.

**Conclusions:**

This systematic review of literature published between 2000 and 2010 identified a range of demographic, clinical, surgical, implant, and provider variables associated with the risk of revision following primary THA. These findings can inform discussions between surgeons and patients relating to the risks and benefits of undergoing total hip arthroplasty.

## Background

Total hip arthroplasty (THA) is a highly successful and cost-effective intervention for addressing pain and functional deficits in patients with advanced hip disease [1-3]. Although the results of THA are generally excellent, some prostheses eventually fail. In many such cases, revision surgery is performed. As a result, revision THA is often used as a proxy for implant failure. In the US, over 50,000 revision THAs are performed every year at a direct cost exceeding $1 billion [4]. Due in part to continued growth in the utilization of primary THA, the number of revisions may increase substantially in the coming decades [5].

Long-term population-based studies have documented rates of THA failure of 1% per year or less, though there is considerable variation in revision rates among patient groups defined by factors such as age and sex [6]. Identifying risk factors for arthroplasty failure is challenging because revision arthroplasty is a relatively infrequent outcome that often occurs a decade or more after the primary procedure. Thus, individual studies require large sample sizes and/or lengthy follow-up periods to detect statistically and clinically significant differences in the risk of revision associated with purported risk factors. Several articles have identified risk factors for revision of primary THA based on data from registries or individual centers, and these findings represent a potential wealth of evidence on the effects of a range of risk factors on revision of primary THA. However, a systematic review is required to aggregate this rich reservoir of evidence and identify relevant trends.

Previous systematic reviews on risk factors for failure of THA have typically focused on specific research questions such as the impact of anesthesia type, [7] underlying diagnosis, [8] or cemented vs. uncemented fixation [9]. A 2008 review by Santaguida et al. examined the effect of patient demographic factors on THA outcome [10]. That review focused on studies published between 1980 and 2001, however, thereby excluding more recently published work that incorporates longer-term follow-up and more contemporary processes of care such as modern cementing techniques, biomaterials, and rehabilitation approaches. To the best of our knowledge, there have been no efforts to aggregate more recent data on a more comprehensive set of risk factors.

The purpose of this systematic review is to summarize the published literature on risk factors for revision of primary THA relating to pre-operative patient demographic and clinical factors, surgical factors (including features of the implant), and health care provider characteristics. In addition to overall revision, we examine risk factors for the three most common indications for revision: aseptic loosening, infection, and dislocation, each of which accounts for at least 15% of total revisions [11-13]. This systematic review may help surgeons more accurately assess the factors associated with failure of THA, and may promote fully informed conversations about the risks and benefits of the procedure between surgeons and patients considering THA.

## Methods

### Search strategy

We conducted a PubMed search on November 16, 2010 to identify studies written in English published between January 1, 2000 and November 1, 2010. We excluded articles published prior to 2000 to ensure that the studies considered reflected secular changes that may have taken place since the only previously published literature synthesis [10]. We restricted the review to articles published in peer-reviewed journals to ensure methodological oversight. We combined PubMed hip arthroplasty MeSH Term keywords with search terms relating to revision and failure risk to produce the following search query:

"("Arthroplasty, Replacement, Hip*"[MeSH Major Topic] OR (total[Title/Abstract] AND hip[Title/Abstract] AND (arthroplasty[Title/Abstract] OR replacement[Title/Abstract])) AND ("risk factor" OR "risk of failure" OR "risk of revision" OR "rate of failure" OR "rate of revision" OR "revision risk" OR survival)"

We performed three levels of screening to identify papers for our review. We reviewed the titles in the search results based on the exclusion criteria outlined in Table 1. We retrieved and reviewed abstracts for all articles deemed potentially eligible at the title stage. Articles that passed the abstract screening were retrieved as full manuscripts for the final level of screening.

**Table 1:** Exclusion criteria for the title, abstract, and paper-level screening of articles retrieved from the PubMed search

**Exclusion criteria, listed in hierarchical order**	**Example or explanation**
• Focus on wrong procedure	*e.g. total knee arthroplasty*
• Focus on risk factors for primary THA	*as opposed to risk of revision of THA*
• Focus on outcomes of revision THA surgery	*as opposed to risk of revision of primary THA*
• No living human subjects	*e.g. canine or simulation study*
• Focus on non-standard THA	*e.g. hip resurfacing*
• Focus on THA to address hip fracture	*as opposed to THA for hip arthritis*
• Focus on non-prosthesis THA outcome	*e.g. mortality*
• Focus on prosthesis-related non-revision outcome	*e.g. polyethylene wear*
• Focus on stem or cup failure only*	*as opposed to revision of any component*
• Focus on specific brand comparison only^+^	*no other comparisons reported*
• Sample has less than 2,500 person-years	*at the title level, case studies were excluded*
• Study includes no new clinical data*	*e.g. literature review*
• Study does not report a comparative risk metric^+^	*e.g. no odds ratio or multiple survival curves*
• Article could not be retrieved^+^	*not available in electronic or print archives*

* Assessed at the abstract and paper levels of screening only.

^+^ Assessed at the paper level of screening only.

To ensure comparability across studies and generalizability, we excluded articles describing samples that included less than 2,500 person-years of follow-up. Given a revision risk of 1% per year, this criterion ensures at least 25 ‘events’ (revisions) per study. Similarly, we required a minimum of 25 cases in case–control studies.

### Validation

A second reviewer independently screened random samples of 200 titles, 100 abstracts, and 40 papers. Any disagreements in the decision of whether to exclude the titles, abstracts, or papers were adjudicated by the senior author (JNK). With the exception of one abstract, the senior author’s assessment of exclusions on all screened items agreed with that of the primary reviewer (JJZP) (the abstract was found to be ineligible at the paper level).

We performed a second validation analysis on the overall screening process, determining agreement on a single set of 200 papers that passed through all three levels of screening (title, abstract, paper). Again, disagreements were adjudicated by the senior author, and here too, the senior author’s assessment concurred with that of the primary reviewer. Finally, the senior author abstracted key data elements on eight eligible papers, blinded to the primary reviewer’s abstraction, and agreed with the primary reviewer’s assessment on all elements in all eight papers.

### Data abstraction

For articles that were eligible for inclusion, we extracted information on study design, sample details, and average follow-up time. We also abstracted data on the particular revision endpoint being studied (revision for any reason, aseptic loosening, infection, or dislocation), the specification of the risk factor being examined (e.g. age dichotomized or defined as a continuous variable), the effect measures (e.g. risk ratio, odds ratio, difference in proportion), confidence intervals, and p-values. We noted instances in which studies indicated in the text that a particular factor was associated with a risk of revision and whether the association reached statistical significance, even if no quantitative metric was provided.

### Analysis

The heterogeneous nature of the studies’ follow-up times, risk factor specifications, and effect measures precluded a formal meta-analysis. Instead, we identified all risk factors that were examined by at least three studies for a given endpoint and described the number and direction of significant associations (defined as p≤0.05 or non-overlapping confidence intervals). We recognize that categorizing studies on the basis of statistical significance of the association of interest has many limitations. For example, this approach does not incorporate the magnitude of association, potential confounding, or study quality. However, it does provide a common framework with which each study can be assessed, providing a broad snapshot of the state of the literature. The findings are intended to identify areas of discordance, which should prompt closer examination of the literature, and of consistency, reflecting findings that appear to be robust across multiple reports.

For some studies that did not include quantitative results in the text, we were able to read results from survival curves presented in figures. In other cases, studies collected data on risk factors but did not present them in a format consistent with our analysis. For these papers, we used the reported frequencies of exposure and revision to compute crude relative risk estimates, along with 95% confidence intervals and p-values. In two instances [13,14], un-stratified frequencies were not reported in the published paper, so we obtained the data from the corresponding author. Statistical analyses were performed using SAS software, Version 9.2 of the SAS System for Windows. (SAS Institute Inc., Cary, NC, USA.)

## Results

### Screening results

**Figure 1:**
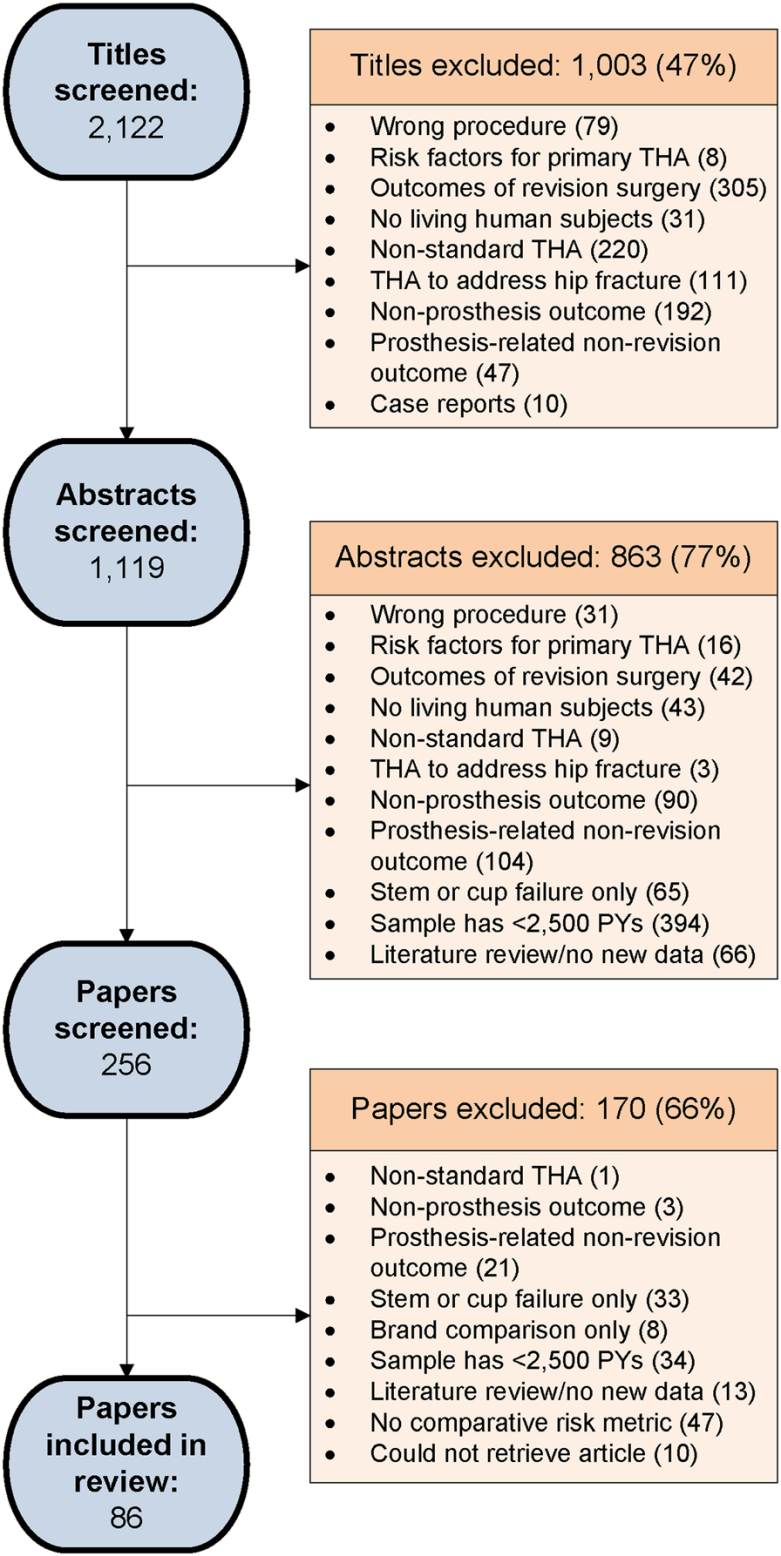
Manuscript search and selection process.

Full search results are represented in Figure 1. Of 2,122 titles identified by our search, we included 86 papers in this review. Of those 86 papers, 65 examined risk factors for revision for any reason, 30 examined risk factors for aseptic loosening, nineteen for infection and twelve for dislocation. In addition, three papers examined unique endpoints: revision for acetabular osteolysis, revision within 90 days of primary THA, and revision for any reason other than infection (which conflates aseptic loosening and dislocation). These papers were not included in the analysis. The full abstraction of study design features and quantitative results on all 86 publications is available upon request. Frequencies for specific risk factors by endpoint are presented in Table 2. Reference numbers for the papers examining each risk factor for each endpoint are provided in Table 3.

**Table 2:** Number of studies reporting on associations between potential risk factors and revision of primary THA

**Specific endpoint**	**Revision (any)**		**Aseptic loosening**		**Infection**		**Dislocation**	
Total for each endpoint*	65		30		19		12	
**Risk Factor**	**N^ (%)**^**+**^		**N (%)**		**N (%)**		**N (%)**	
	**Demographic Factors**							
Age	26	(40%)	12	(40%)	5	(26%)	4	(33%)
Sex	18	(28%)	10	(33%)	6	(32%)	2	(17%)
BMI/body weight	5	(8%)	2	(7%)	2	(11%)	1	(8%)
Race	1	(2%)	—		1	(5%)	—	
Socioeconomic status	1	(2%)	—		1	(5%)	—	
Tobacco use	—		1	(3%)	—		—	
Alcohol use	—		—		1	(5%)	—	
Preparation of living aids	—		1	(3%)	—		—	
	**Clinical Factors**							
Underlying diagnosis	21	(32%)	8	(27%)	6	(32%)	3	(25%)
Comorbidity (Charlson)	5	(8%)	—		2	(11%)	—	
Diabetes	1	(2%)	1	(3%)	2	(11%)	1	(8%)
Medication use	3	(5%)	4	(13%)	3	(16%)	3	(25%)
Bilateral vs. unilateral THA	3	(5%)	—		—		—	
Genetic predisposition	—		1	(3%)	2	(11%)	—	
Prior hip surgery	1	(2%)	1	(3%)	1	(5%)	—	
Post-THA childbirth	1	(2%)	—		—		—	
	**Surgical and Implant-Related Factors**							
Fixation	11	(17%)	6	(20%)	6	(32%)	2	(17%)
Femoral head size	3	(5%)	2	(7%)	—		3	(25%)
Bearing	1	(2%)	1	(3%)	—		1	(8%)
Coating	1	(2%)	1	(3%)	—		—	
Femoral head material	1	(2%)	—		—		—	
Other design-related factors	1	(2%)	2	(7%)	1	(5%)	—	
Operating time	2	(3%)	1	(3%)	4	(21%)	2	(17%)
Surgical approach	1	(2%)	1	(3%)	1	(5%)	3	(25%)
Ventilation	1	(2%)	—		2	(11%)	—	
Anesthesia	—		—		2	(11%)	—	
Infection prophylaxis	1	(2%)	1	(3%)	1	(5%)	—	
Fixation technique	1	(2%)	1	(3%)	—		—	
Units blood transfused	—		—		1	(5%)	—	
	**Health Care Provider-Related Factors**							
Hospital volume	6	(9%)	1	(3%)	—		—	
Hospital teaching status	2	(3%)	—		1	(5%)	—	
Rural vs. urban hospital	—		—		1	(5%)	—	
Surgeon volume	4	(6%)	—		—		—	
Surgeon experience	2	(3%)	—		—		—	

* Papers that reported on multiple endpoints are included in each of the relevant columns.

^ — indicates no papers examined the risk factor for that endpoint.

+ Column percentages, not mutually exclusive.

**Table 3:** Reference numbers for papers examining each risk factor for each endpoint

**Risk factor**	**Reference numbers for papers**			
	**Revision (any)**	**Aseptic loosening**	**Infection**	**Dislocation**
	**Demographic Factors**			
Age	[1,13-37]	[1,13,16,17,19,20,28,38-42]	[13,43-46]	[26,47-49]
Sex	[13,18-22,27,29,31-35,37,50-53]	[1,13,17,19,20,28,38,40-42]	[13,43-46,54]	[47,48]
BMI/body weight	[55-59]	[39,55]	[44,57]	[57]
Race	[34]	—	[45]	—
Socioeconomic status	[15]	—	[45]	—
Tobacco use	—	[60]	—	—
Alcohol use	—	—	[44]	—
Preparation of living aids	—	[40]	—	—
	**Clinical Factors**			
Underlying diagnosis	[13,14,16,18,21,27,31,35,37,47,51,53,61-69]	[1,13,16,28,38,40,64,69]	[13,43,44,46,54,61]	[47,48,61]
Comorbidity (Charlson)	[27,29,33-35]	—	[45,70]	—
Diabetes	[70]	[70]	[44,70]	[70]
Medication use	[68,71,72]	[60,68,71,72]	[68,71,72]	[68,71,72]
Bilateral vs. unilateral THA	[73-75]	—	—	—
Genetic predisposition	—	[76]	[77,78]	—
Prior hip surgery	[18]	[28]	[46]	—
Post-THA childbirth	[79]	—	—	—
	**Surgical and Implant-Related Factors**			
Fixation	[13,17,19,25,26,52,61,80-83]	[13,17,19,81-83]	[13,26,43,46,81,83]	[47,83]
Femoral head size	[50,51,84]	[41,42]	—	[47-49]
Bearing	[85]	[86]	—	[49]
Coating	[19]	[19]	—	—
Femoral head material	[51]	—	—	—
Other design-related factors	[87]	[87,88]	[43]	—
Operating time	[33,83]	[83]	[43,45,46,83]	[48,83]
Surgical approach	[11]	[11]	[11]	[11,26,48]
Ventilation	[89]	—	[43,46]	—
Anesthesia	—	—	[44,46]	—
Infection prophylaxis	[89]	[89]	[89]	—
Fixation technique	[90]	[90]	—	—
Units blood transfused	—	—	[44]	—
	**Health Care Provider-Related Factors**			
Hospital volume	[20,21,35,37,91,92]	[20]	—	—
Hospital teaching status	[21,35]	—	[45]	—
Rural vs. urban hospital	—	—	[45]	—
Surgeon volume	[26,29,35,92]	—	—	—
Surgeon experience	[21,93]	—	—	—

We summarize the data on risk factors that were examined in at least three papers for at least one endpoint in Table 4. Specifically, we indicate the number of papers that found a statistically significant increased or decreased risk and the number of papers that found no significant association. Risk factors examined by at least three papers for which no significant associations were reported are not discussed in the text, but are included in Table 4.

**Table 4:** Comparison of risk factors for revision for all endpoints addressed by at least three studies

**Risk factor**	**Studies reporting on a risk factor for each endpoint: total number, number reporting a statistically significant (p≤.05) increased (+) or decreased (**—**) risk of revision, and number with no significant association (p>.05)**															
	**Revision (any)**				**Aseptic loosening**				**Infection**				**Dislocation**			
	**N**	**p≤.05**		**p>.05**	**N**	**p≤.05**		**p>.05**	**N**	**p≤.05**		**p>.05**	**N**	**p≤.05**		**p>.05**
		**+**	**—**			+	—			+	—			+	—	
**Demographic and Clinical Factors**																
Young age	*26:*	15	2	9	*12:*	8	0	4	*5:*	0	0	5	*4:*	0	2	2
Male sex	*18:*	7	1	10	*10:*	5	1	4	*6:*	5	0	1	—			
Higher BMI/weight	*5:*	0	0	5	—				—				—			
Higher Charlson score	*5:*	5	0	0	—				—				—			
**Underlying Diagnosis**																
RA (vs. OA)	*9:*	3	0	6	*3:*	0	1	2	*6:*	1	0	5	*3:*	1	0	2
AVN (vs. OA)	*8:*	5	1	2	—				*3:*	1	0	2	—			
RA (vs. AVN)	*4:*	0	0	4	—				*3:*	0	0	3	—			
DDH (vs. RA)	—				—				—				*3:*	—	0	3
**Surgical and Implant-Related Factors**																
Uncemented (vs. cem)	*10:*	5	2	3	*6:*	3	3	0	*4:*	0	1	3	—			
Hybrid (vs. cem)	*4:*	2	0	2	—				—				—			
Hybrid (vs. uncem)	*4:*	1	2	1	—				—				—			
Larger femoral head	*3:*	2	0	1	—				—				*3:*	0	3	0
Uncem (vs. cem AL)*	—				—				*3:*	1	0	2	—			
Cem NA (vs. uncem)	—				—				*3:*	3	0	0	—			
Cem NA (vs. cem AL)	—				—				*3:*	3	0	0	—			
Longer operating time	—				—				*4:*	3	0	1	—			
Posterior surgical approach (vs. lateral)	—				—				—				*3:*	2	0	1
**Health Care Provider-Related Factors**																
Lower hospital volume	*6:*	1	0	5	—				—				—			
Lower surgeon volume	*4:*	3	0	1	—				—				—			

* Cem AL = antibiotic-loaded cement.

Cem NA = cement not containing antibiotics.

In order to present a consistent assessment, we required at least three papers to address the same pairwise comparison to meet inclusion for Table 4. Several underlying diagnoses met this threshold for at least one comparison. Almost all of these comparisons used OA as the reference group, so where possible, pairwise comparisons among the other diagnoses were performed using the statistical method outlined above.

### Revision for any reason

#### Demographic and clinical factors

##### Age

Of the 26 papers [1,13-37] that examined age at primary THA as a risk factor for revision for any reason, seventeen reported a statistically significant association with revision risk. Fifteen reported an *increased risk of revision for younger patients*, irrespective of the particular age categories examined (categories ranged from ≤40 to ≥85). The risk of revision generally decreased per additional decade of age. Two studies reported a statistically significant *increased risk of revision for older patients.*

##### Sex

Eight of the eighteen papers [13,18-22,27,29,31-35,37,50-53] that evaluated the association of sex with risk of revision reported a statistically significant association. Seven reported *an increased risk for men* and one study (6%) reported an *increased risk of revision for women.*

##### Comorbidity (Charlson score)

All five papers [27,29,33-35] that examined comorbidities found a statistically significant *higher risk of revision for a higher Charlson comorbidity score.*

#### Underlying Diagnosis

##### Rheumatoid arthritis (RA) vs. osteoarthritis (OA)

Of the nine papers [13,21,27,37,47,61-64] that compared RA and OA patients who underwent THA, three reported a statistically significant *increased revision risk for RA*.

##### Avascular necrosis (AVN) vs. OA

Eight papers [13,14,18,27,47,53,61,65] compared patients with AVN and OA, six of which reported a statistically significant association. Five papers reported an *increased risk for AVN patients* and one paper found a *decreased risk for AVN patients.*

##### RA vs. AVN

*No statistically significant associations* were reported in the four papers [13,27,47,61] that compared the revision risks in subjects with RA and AVN. Hailer et al. [13] reported an increased risk for AVN patients in their adjusted results, but we were unable to establish the statistical significance of this comparison. The papers comparing RA with AVN did not indicate whether the cases of AVN might have been accompanied by secondary OA.

#### Surgical and implant-related factors

##### Uncemented vs. cemented fixation

Of the ten papers [13,17,19,25,26,52,80-83] that considered uncemented vs. cemented fixation, seven reported a statistically significant association with revision risk. Five papers reported a statistically significant *increased risk for uncemented prostheses* and two found a significant *increased risk of revision for cemented prostheses*.

##### Hybrid vs. cemented fixation

Four papers [25,26,52,82] compared hybrid (cemented stem and uncemented cup) and fully cemented fixation, and two of them found a statistically significant *increased risk of revision for hybrid THA.*

##### Hybrid vs. uncemented fixation

Of the four papers [25,26,52,82] comparing hybrid (cemented stem and uncemented cup) and fully uncemented fixation, three papers reported a statistically significant association. Two papers found an *increased risk of revision for uncemented prostheses* and one found an *increased risk for hybrid prostheses*.

##### Femoral head size

Two of the three papers [50,51,84] examining femoral head size found a statistically significant *increased risk of revision for any reason for larger head size*. In both studies, 28mm heads were the largest head size examined.

#### Health care provider-related factors

##### Hospital volume

Six papers [20,21,35,37,91,92] reported on the association of hospital volume with revision. One study found a statistically significant *increased risk for low-volume hospitals*.

##### Surgeon volume

Of the four papers [26,29,35,92] that considered surgeon volume, three found a statistically significant *increased risk of revision for low-volume surgeons.*

### Revision for aseptic loosening

#### Demographic and Clinical Factors

##### Age

Of the twelve papers [1,13,16,17,19,20,28,55-59] that evaluated the association of age at primary THA with the risk of revision for aseptic loosening, eight papers reported a statistically significant *increased risk for younger patients.* While these studies used different age stratifications, they consistently found higher risk in younger patients.

##### Sex

Ten papers [1,13,17,19,20,28,55,57-59] considered the association of sex with revision for aseptic loosening, and six papers reported a statistically significant association. Five papers reported an *increased risk for men* and one paper found an *increased risk for women*.

#### Underlying diagnosis

##### RA vs. OA

Three papers [1,13,64] compared the risk of revision for aseptic loosening for patients with RA and OA, and one paper found a statistically significant *increased risk for OA patients*.

#### Surgical and implant-related factors

##### Uncemented vs. cemented fixation

All six papers [13,17,19,81-83] reporting on fixation found statistically significant associations with risk of revision for aseptic loosening. Three papers found an *increased risk for fully uncemented prostheses* and the other three found an *increased risk for cemented prostheses*.

### Revision for infection

#### Demographic and Clinical Factors

##### Sex

Five of the six papers [13,43-46,54] that examined sex as a risk factor for revision for infection found a statistically significant *increased risk for men*.

#### Underlying Diagnosis

##### RA vs. OA

Six papers [13,43,44,46,54,61] compared the risk of revision for infection for patients with RA and OA (including two papers that compared OA and “inflammatory arthritis”), one of which found a statistically significant *increased risk for patients with RA*.

##### AVN vs. OA

Of the three papers [13,44,46] that compared patients with AVN and OA for risk of revision due to infection, one paper reported a significant *increased risk for AVN*.

#### Surgical and Implant-Related Factors

##### Uncemented vs. cemented fixation

Four papers [13,26,81,83] reported on uncemented vs. cemented fixation as a risk factor for revision due to infection. One paper found a statistically significant *increased risk for cemented fixation*.

##### Uncemented vs. antibiotic-loaded cement

Of the three papers [43,46,81] that compared uncemented prostheses with those containing antibiotic-loaded cement, one found a statistically significant *increased risk for uncemented prostheses*.

##### Uncemented vs cement not containing antibiotics

All three papers [43,46,81] that compared revision risk due to infection for uncemented prostheses vs. those with cement not containing antibiotics found a statistically significant *increased risk for cement without antibiotics*.

##### Antibiotic-loaded cement vs. cement not containing antibiotics

All three papers [43,46,81] that compared the risk of revision for infection between prostheses cemented with and without antibiotics found a statistically significant *increased risk for cement not containing antibiotics.*

##### Operating time

Of the four papers [43,45,46,83] that examined operating time as a risk factor for revision due to infection, three found a statistically significant *increased risk for longer surgeries*.

### Revision for dislocation

#### Demographic and Clinical Factors

##### Age

Four papers [26,47-49] examined age at primary THA as a risk factor for revision due to dislocation, two of which found a statistically significant *increased risk for older age*.

#### Underlying Diagnosis

##### RA vs. OA

Of the three papers [47,48,61] that compared the risk of revision for dislocation in patients with RA and OA, one paper reported a significant *increased risk for patients with RA*.

#### Surgical and Implant-Related Factors

##### Femoral head size

All three papers [47-49] that examined femoral head size as a risk factor for revision due to dislocation found a statistically significant *increased risk for smaller head size*. In all three studies, head sizes >28mm were associated with a lower risk than head sizes ≤28mm.

##### Posterior vs lateral surgical approach

Of the three papers [11,26,48] that examined the risk of revision for dislocation associated with the posterior vs. lateral surgical approach, two found a statistically significant *increased risk for the posterior approach*. Of note, the third study reported that the posterior approach was associated with a “greater early risk of dislocation,” but that the failure rates for the two approaches evened out later on in the seven-year follow-up period [26].

## Discussion

We conducted a systematic literature review on risk factors for revision of primary THA relating to the characteristics of the patient, surgery, implant, and health care provider. We focused on papers published since the year 2000 in order to provide the most up-to-date findings. We present information on risk factors studied in at least three papers for one of our endpoints of interest: revision for any reason or for an indication of aseptic loosening, infection, or dislocation.

The potential risk factors that were studied by at least three papers for several endpoints were patient age and sex, underlying diagnosis, and implant fixation (cemented vs. uncemented). Younger age at the time of primary THA was generally associated with a *higher* risk of overall revision and revision for aseptic loosening, but younger age was also associated with a *lower* risk of dislocation. Male sex was generally associated with a higher risk of revision for aseptic loosening and infection, and had a less consistent association with a higher risk of overall revision.

Our findings with regard to fixation were inconclusive. Consistent with prior reviews, [6,9] we found a trend toward an increased revision risk for fully uncemented prostheses: often studies that examined fixation as a risk factor for overall revision, five found a statistically significant increased risk for uncemented prostheses, two found a statistically significant increased risk for cemented prostheses, and three reported no significant associations. A review by Morshed et al. [9] suggests that the performance of fully uncemented implants may be improving over time as compared with cemented implants. Our findings suggest the opposite: among papers included in this review, the two that found an increased risk for cemented implants examined primary THAs implanted in the 1980s and early 1990s, while studies focusing on more recently implanted THAs found a higher revision risk for fully uncemented implants. This discrepancy may be due to the fact that nine of the 20 papers in the Morshed review were published prior to 2000 and were therefore excluded from our initial search query. Many of the rest were ineligible for this review because they had small sample sizes or examined failure of a specific component rather than revision of any component.

There were other notable findings concerning risk factors for specific endpoints as well. Greater comorbidity and low surgeon volume were both associated with a higher risk of overall revision, as was a diagnosis of AVN as compared to OA. Longer operating time was associated with a higher risk of revision for infection. Smaller femoral head size was associated with an increased risk for dislocation.

The results of this review indicate that factors may increase the risk of revision for certain endpoints but be protective for others. For example, younger age was generally associated with an increased risk of revision for aseptic loosening and for overall revision, but seemed to reduce the risk of dislocation. Similarly, smaller femoral head size increased the risk of dislocation, but may also be associated with a decreased risk of overall revision. In cases where a given risk factor works in different directions for the specific indications, we might expect less clarity and uniformity with regard to overall revision.

Several challenges and limitations of our review bear comment and suggest avenues for further work. The studies in our review examined revision surgery as an endpoint, but revision rates do not capture failed implants that are not surgically revised. Focusing on revision misses patients with painful prostheses who do not seek medical attention, who choose not to have revision, or who are not offered revision because their general health makes them unsuitable surgical candidates. Thus, revision is a specific but insensitive marker of THA failure. Validation studies on the Swedish Register have indicated that clinical failure rates at ten years, as defined by radiographic loosening in combination with the Harris Hip Score and the Western Ontario and McMaster Osteoarthritis Index (WOMAC), are at least twice as high as the revision rates reported by the Register [3,94,95].

With the exception of case–control studies, we limited our review to large studies with at least 2,500 person-years of follow-up to enhance the generalizability of our findings and the stability of the estimates from the included studied. Eleven case–control studies were included in the review, but only two reported on risk factors that were included in Table 4. Approximately half the papers included in our review were retrospective analyses of data collected in population-based registries. This may account for the relative paucity of studies reporting on factors that are less commonly tracked in registries, such as socioeconomic status, genetic factors, functional status, and features of the operating room. This observation underscores the value of comprehensive arthroplasty registries for research purposes, above and beyond their role in identifying prostheses or patient populations with unusually high rates of failure or complications [96,97].

In addition to the person-years exclusion of smaller cohort studies or studies with shorter follow-up, our results may have been influenced by publication bias favoring statistically significant results. We are only able to summarize the data that authors chose to include in their published papers, and although some authors report all the results of their analyses, others may choose to report only those findings they consider most interesting or relevant.

The use of statistical significance as a binary outcome represents another potential limitation. Statistical significance cut-offs are inherently arbitrary, and though they can provide a useful guide for identifying consistent patterns, they are directly tied to sample size and duration of follow-up [98]. Table 4 allows us to see risk factors with a clear signal across papers, but without also considering the range of reported effect sizes, we cannot determine if the increased or decreased risk is clinically meaningful.

Due to the large number and variety of papers and potential risk factors examined in this review, we were unable to score each study for methodological quality, contrast design features, or compare quantitative results. Listing a tally of positive, negative, and non-statistically significant associations is “rough justice” with respect to any particular factor, but it can serve as a guide to the overall state of research on revision of THA. This review provides a comprehensive picture of what has and has not been examined in larger clinical studies, as well as a sense of whether the findings have been concordant across studies. Researchers may find it helpful to see what has already been done, and where there are opportunities for further work to deepen or clarify our understanding of certain issues.

Finally, we call attention to the importance of adjusting for potential confounders. In some of the studies in this review, the unadjusted and adjusted results for certain risk factors produced statistically significant associations in opposite directions due the presence of confounding factors [13]. For example, patients with high BMI undergoing THA tend to be younger than the average patient, so a failure to adjust for the effect of young age could result in an inflated risk estimate for BMI. In this review, we abstracted and reported adjusted results whenever possible. Many papers provided only unadjusted results, however, and we were unable to adjust for confounders when calculating risk ratios from raw data.

## Conclusions

In this review, factors found to be consistently associated with revision included younger age, greater comorbidity, a diagnosis of AVN as compared to OA, low surgeon volume, and larger femoral head size. Male sex was associated with revision due to aseptic loosening and infection. Longer operating time was associated with revision due to infection. Smaller femoral head size was associated with revision due to dislocation.

These findings may be useful to surgeons and patients contemplating THA or living with THA as they discuss the scientific evidence for potential risk factors for revision. Some important factors, such as prosthesis materials and design, were not addressed by a sufficient number of papers that met our criteria to be included in this review, and the results for factors like fixation did not produce a clear signal. Further research could clarify the prognostic effect of these factors. We also need more work to determine the level of agreement between risk factors for *revision* of primary THA, as identified in this review, and risk factors for *failure* of primary THA, a more pertinent outcome for patients, but one that is much more difficult to study.

## Abbreviations

AVN: Avascular necrosis; DDH: Developmental dysplasia of the hip; OA: Osteoarthritis; RA: Rheumatoid arthritis; THA: Total hip arthroplasty; WOMAC: Western Ontario and McMaster Osteoarthritis Index.

## Competing interests

The authors declare that they have no competing interests.

## Authors’ contributions

JJZP, EL, and JNK conceived and designed the review. JJZP performed the search, screening, and abstraction, and JNK provided validation (see Validation in Methods). RLB performed the data analysis. All authors were involved in drafting and revising the manuscript, and all gave approval of the final version.

## Grant support

NIH/NIAMS P60 AR 47782, K24 AR 057827, T32 AR 055885

## Pre-publication history

The pre-publication history for this paper can be accessed here:

http://www.biomedcentral.com/1471-2474/13/251/prepub
